# Effect of Repeated Plant Debris Reutilization as Organic Amendment on Greenhouse Soil Fertility

**DOI:** 10.3390/ijerph182111544

**Published:** 2021-11-03

**Authors:** Francisco José Castillo-Díaz, José Ignacio Marín-Guirao, Luis Jesús Belmonte-Ureña, Julio César Tello-Marquina

**Affiliations:** 1 Department of Agronomy, Research Centre for Mediterranean Intensive Agrosystems and Agrifood Biotechnology, University of Almería, 04120 Almería, Spain; Franjcd95@hotmail.com (F.J.C.-D.); jtello@ual.es (J.C.T.-M.); 2 Department of Sustainable Plant Protection, Institute for Research and Training in Agriculture and Fisheries, 04745 Almería, Spain; josei.marin@juntadeandalucia.es; 3 Department of Economy and Business, Research Centre for Mediterranean Intensive Agrosystems and Agrifood Biotechnology, University of Almería, 04120 Almería, Spain

**Keywords:** circular economy, bioeconomy, waste management, tomato crop, agriculture, organic fertilizer

## Abstract

Greenhouse agriculture typically generates large amounts of waste with plant residue (agricultural biomass) being the most abundant. This residue is generated on a seasonal basis, which complicates the external management of the material. Recently, the European Union (EU) has been implementing a policy based on sustainability through the circular economy that seeks to minimize waste generation. The effect of reusing 3.5 kg·m^−2^ tomato plants from the previous season as the only fertilizer versus no fertilization and inorganic fertilization in 215-day tomato cycles after transplanting was studied in this trial. The study was carried out during three seasons in greenhouse agriculture in Almeria (Spain) with the repeated use of the solarization technique. The plant debris had similar production results during two of the three seasons and fruit quality parameters were similar to inorganic fertilization. In addition, some physicochemical variables improved and the biological depressive effect of solarization was mitigated. The results suggest that the reuse of the tomato plant debris as the only fertilizer could be an alternative to conventional fertilization under the conditions tested.

## 1. Introduction

The province of Almeria (Spain) is the part of the world with the highest concentration of greenhouse surface [1,2,3,4]. The implementation of this intensive agricultural production system has increased the productivity and profitability of its crops in just 60 years [5] while transforming Almeria into one of the major suppliers of fruit and vegetable products in the EU. The agricultural development of the area has enriched the socioeconomic structure of the province [5,6]. This is a production system that, due to the climatic conditions of the area and the characteristics of its greenhouses (e.g., Almeria or “Raspa y Amagado” type), does not require climatic correction [7,8]. This fact, along with various agroecological techniques and commonly used cultivation methods (e.g., biological control, grafting, integrated pest and disease management) makes this production system one that requires less energy consumption than other similar agricultural systems [9] and also improves the sustainability of the agrosystem under the production principles of different types of certifications [7]. However, there have also been impacts on area ecosystems (e.g., loss of biodiversity, erosion, overexploitation, and eutrophication of aquifers, etc.) that seriously threaten the environmental sustainability of the production model. This requires the formulation of various corrective measures to reverse the situation [4,10,11]. One of the main causes is inefficient management of agricultural waste. This is an endemic problem within this production system that caused a sanitary crisis at the end of the 20th century that ended up forcing public intervention [4].

European regulations enforce the management of agricultural waste through its transformation into by-products when possible (e.g., livestock feed, bioenergy, organic amendments, substrates, plastics, plastic pellets, etc.) [2,12]. The legal bases are founded on the principles of the circular economy and the bioeconomy, which favor the implementation of EU sustainability strategies applicable throughout its productive agriculture sector [13,14,15,16]. The implementation of these strategies is one of the collective changes to be made by the Almeria Model [10], which presents abundant opportunities to apply the principles of the circular economy in its production phases [4,17].

Plant debris (agricultural biomass) are considered a wasted by-product in some European agricultural systems [15]. The location and seasonality of their production, as well as a lack of space on some farms, inadequate transport logistics, the mixing of plant debris with plastic trellising inputs, and the poor phytosanitary condition of the material make its management difficult [18]. There is also a failure to maintain stable inputs for the transformation processes of the plant element [17], which does not justify the investment in building external treatment centers in certain locations while also transport costs increase [18]. In the Almeria model, 1.8 million tons of plant debris are generated annually, 80% of which is generated in only three months (February, May, and June) [18]. Some of the alternatives evaluated to mitigate this problem (transformation into bioenergy or animal feed) do not offer a viable option compared to the predominant management of the by-product [4,19], which currently consists of its delivery to an agent authorized by the administration to transform the plant material into compost [18]. However, several studies have posited self-management of plant debris by farmers as a suitable reuse process [4,7,17,20,21,22]. This is a great opportunity for the Almeria Model to apply the principles of the circular economy and the bioeconomy, which so far have not been extensively implemented in Almeria greenhouse agriculture [7,17,18]. Through this management methodology, it is possible to generate economic [4] and productive [20,21,22] benefits since its use as an organic amendment makes it possible to reduce and even eliminate external inputs of fertilizing materials during the crop cycle [20,21] thanks to the mineral elements associated with these plant by-products [23].

This material can also be used to improve soil fertility [24,25,26], which is defined by its physical, chemical, and biological components [27]. Specifically, the addition of organic amendments has a positive influence on these components even when their introduction is carried out through the solarization technique [20,28,29,30,31,32]. This soil biodisinfection protocol combines the effects of solarization [33] and biofumigation [34] and is traditionally used as an alternative to chemical control of soil pathogens [35,36,37,38]. Thus, the biological component is considered essential for maintaining and improving the health and fertility of agricultural soil [39]. It is, therefore, essential to support actions to protect soil biodiversity and promote its sustainable use and management through the application of sustainable practices [40,41].

Previous research has addressed the study of the incorporation of plant debris of different origins into the soil with subsequent solarization on agronomic variables and also on edaphic variables that determine the health and fertility of the soil, although normally the studies address these variables in isolation and/or under different conditions. However, the information obtained from the study of specific plant material on all of these variables is scarce. This information would help the practice of self-management of plant debris in the Almeria Model in accordance with the principles of the circular economy. This would contribute to the sustainability of agrosystems while providing a solution to the problem that the management of this material has posed up until now. Therefore, the objective of this research was to evaluate the effect of the repeated incorporation of tomato plant debris into the soil with subsequent solarization as the only nutrient source during three lengthy tomato production cycles on several variables, including production, crop quality, physical, chemical, and biological qualities of the soil, and also the vigor of tomato and cucumber seedlings grown under controlled conditions.

## 2. Materials and Methods

### 2.1. Location, Climate and Greenhouse

The trials were conducted during three consecutive years (2015–2016, 2016–2017, and 2017–2018 seasons) in the facilities of the UAL-ANECOOP Experimental Farm located in the province of Almeria (Spain), the largest Mediterranean greenhouse growing region and the main greenhouse tomato production area in the EU. The experimental greenhouse was representative of the Mediterranean “Raspa y amagado” greenhouse [8] with a maximum and minimum height of 4.70 and 3.40 m, respectively. The greenhouse cover was made of 200 µm thick transparent polyethylene, with zenithal side windows, which included an anti-strip mesh. The greenhouse had a surface area of 1784 m^2^ and a northwest-southwest orientation as well as the crop rows. The irrigation system consisted of two totally independent sectors. The nominal flow rate of the emitters used was 3 L·h^−1^. The greenhouse soil consisted of a mixture of gully soil and sand. At the beginning of the study, the soil had 8.8 ± 6.2% of clay, 76.0 ± 4.1% of sand, and 7.0 ± 0.8% of silt. The soil pH was 7.80 ± 0.22, the organic matter content 0.93 ± 0.14%, the carbon/nitrogen (C/N) ratio 7.0 ± 0.8, the amount of active limestone 3.9 ± 1.5%, the amount of carbonates of 26.8 ± 3.1% and the values of primary macronutrients (N/P/K) of 0.078 ± 0.014% N, 79.00 ± 10.98 mg·kg^−1^ P, and 259,29 ± 162,08 mg·kg^−1^ K. The soil had been free of edaphic diseases during the previous two years [20].

### 2.2. Cultivation, Experimental Design, and Description of Treatments

Three consecutive winter tomato cycles were undertaken, with a duration of 215, 212, and 217 days after transplanting (DAT), respectively. Transplanting was carried out during the first week of September in each of those three years. The tomato varieties used were (*Solanum lycopersicum* Mill.) and “Pitenza F1” (Enza Zaden, Enkhuizen, The Netherlands) with a planting density of 2 plants/m^2^. Cultural practices were in accordance with the recommendations offered by Camacho-Ferre [42]. The plants were guided with raffia ropes without using trellising clips. Pest and disease control was carried out in compliance with integrated production (IP) regulations [42].

The treatments applied were based on crop nutrition. Three treatments were considered: (1) conventional inorganic fertilization (i.e., IF), based on Steiner’s ideal nutrient solution [43] until reaching an electrical conductivity of 3 dS·m^−1^ (water + nutrient solution); (2) fertilization with fresh tomato plant debris from the previous season at a rate of 3.5 kg·m^−2^ (i.e., PD); and (3) exclusive irrigation water supply without fertilization (i.e., test) (Table 1). The experimental design corresponds to a single-factor design with four replications (n = 4).

Over the course of the three-year study, solarization treatments were applied to the entire surface of the greenhouse during the summer and prior to crop establishment. Previously it was only in the experimental plots of the PD treatment that the remains of tomato plants were applied. To do this, the tomato plants from the previous crop were separated from the raffia used for trellising and deposited on the central concrete aisle of the greenhouse. The tomato plant debris were then crushed with a hammer chopper, which was applied at a rate of 3.5 kg·m^−2^ over the surface of the four experimental plots, and then mixed using a cross and surface pass (20–30 cm deep) with a rotovator. Once the irrigation branches were in place, the entire surface of the greenhouse was covered with transparent polyethylene plastic at a thickness of 50 µm. To prevent the loss of humidity and gases generated during the biodecomposition of organic materials, the polyethylene cover was sealed around the perimeter with a rectangular trench measuring 0.20 m at the base and a height of 0.30 m. The plastic sheets were then joined with staples and the greenhouse posts were sealed with adhesive tape. After all the above steps were completed, consecutive irrigation was applied for four days until the soil reached the saturation point (56 L·m^−2^·year^−1^). The solarization treatments continued from June through September.

The area of the experimental plots covered 40 m^2^ (80 plants each) for the IF and PD treatments and half for the test treatment.

### 2.3. Analyzed Variable

#### 2.3.1. Crop Yield

Various production parameters were calculated from the first harvest at 92 DAT in the first year and at 99 DAT in the second and third years in calculating the point production of each harvest, accumulated production, and fruit weight. The evaluations were carried out during the 14th, 15th, and 18th harvests of each of the three seasons, respectively. Fruit weight was obtained from the measurement of 25 randomly selected fruits in each harvest. For this purpose, a scale (Mettler Toledo, Columbus, OH, USA) with a sensitivity of 0.1 g was used. Harvesting was carried out in accordance with the commercial maturity criteria required by the marketing entity.

#### 2.3.2. Fruit Quality

Tomato fruit quality was evaluated on five occasions in each of the seasons (at 106, 134, 173, 194, and 215 DAT in the first year; at 127, 142, 170, 184, and 198 DAT in the second year; and at 126, 147, 168, 196, and 217 DAT in the third year). For each evaluation, ten tomatoes were selected from each experimental unit analyzing a total of 200 tomatoes from each treatment per year. The variables evaluated included equatorial diameter using a digital caliper with a sensitivity of 0.01 mm (Mitutoyo; Kanagawa, Japan), mean fruit flesh firmness obtained from three equidistant points and on a surface of 0.5 cm^2^ using a durometer with a sensitivity of 0.001 kg·cm^−2^ (Penefel DFT14, Agrosta, Compainville, France), fruit pulp pH with a 0.01 sensitivity pH meter (pH-25, Crison, Barcelona, Spain), and soluble solids content (TSS) in the fruit pulp using a 0.1 brix (pal-1, Atago, Tokyo, Japan).

#### 2.3.3. Evaluation of Soil Variables

##### Sampling and Soil Samples

Soil sampling was carried out at seven different times throughout the study. The first was conducted at the beginning and prior to the application of solarization to determine the initial conditions of the soil. The remaining samples were distributed evenly over the three years of the trial at a rate of two per year, which coincided with the day solarization treatments were completed (first week of September) and at the end of the crop (second week of April).

Soil samples (≈10 kg each) were collected with a shovel at three equidistant points located on the central crop line of each experimental unit. The samples were then mixed and homogenized in a transparent polyethylene bag and kept refrigerated (8 °C) until processing and/or analysis.

##### Analysis of Fungal and Culturable Bacterial Microbiota

- Preparation of soil samples:

Soil samples were subjected to a drying, crushing, and sieving treatment under the recommendations offered by Tello-Marquina et al. [44]. They were placed in plastic trays where they were left to dry at room temperature (20–25 °C) for 7–10 days until constant weight. They were then crushed using a porcelain mortar and the resulting product was sieved with a 200 μm. The instruments used were washed and disinfected by flaming with alcohol between samples.

- Analytical method:

The soil culturable fungal and bacterial microbiota was analyzed by the successive dilutions method [44]. This technique was selected to isolate the live culturable fraction and to allow the study of its functionality [45]. The culture medium used was agar-malt acidified with a 1% citric acid solution to a pH of 4.8 to avoid excessive bacterial growth. Ten subreplicates (i.e., *Petri* dishes) of each soil sample were made at 10^−3^ and 10^−4^ dilutions. The *Petri* dishes (9 cm diameter) were incubated at room temperature (20–25 °C) for 4–7 days. Subsequently, total colony forming units (CFU) of fungi and bacteria were quantified and morphological identification at the genus scale of the fungi found in each *Petri* dish was performed [46,47], eventually expressing the results CFU/g dry soil. For the description of the fungal community structure at the end of cultivation in the three years of study, five classical diversity indices were selected: Simpson’s diversity index [48], Shannon–Wiener’s diversity [49], Margalef’s index [50], Pielou’s Equity index [51] and number of genera.

Fungi of the genus *Fusarium* were isolated using the Warcup technique with a semi-selective culture medium of Komada [52] modified by Tello et al. [44]. Sixteen Petri dishes per sample were used and they were divided into four blocks of four plates. Incubation was performed at room temperature (20–25 °C) for 4–7 days. The total number of CFU’s was quantified and morphological identification was performed at species scale following the taxonomic criteria of Nelson et al. [53] and Leslie and Summerell [54] finally expressing the results CFU/g soil.

##### Physicochemical Analysis

The determination of the physical and chemical parameters of the soil samples was outsourced to the Agroalimentary Laboratory of Granada of the Ministry of Agriculture, Fisheries, and Rural Development of the Junta de Andalucia. The evaluations were carried out using the standardized methods described in Order 5/12/1975 [55] from a soil subsample of 0.5 kg. These evaluations were performed on soil samples from three experimental plots, in the case of the IF and PD treatments, and from one experimental plot in the test treatment.

Soil organic carbon (SOC) was found by oxidation of the element with potassium dichromate in the presence of sulfuric acid using a 0.5 g sample of soil. Subsequently, soil organic matter (SOM) content was estimated from the Waksman factor (i.e., 1.724).

Total soil nitrogen (Nt) was obtained by the Kjeldahl method modified by Olsen from a 5 g sample of soil that had been sieved through a 1 mm beam sieve. Before digestion of the organic nitrogen, a reduction of the nitric form to ammoniacal was carried out to obtain the Nt content. Assimilable phosphorus (P) was calculated through its solubility in sodium bicarbonate from a 5 g soil sample. Assimilable potassium (K) was found from the capacity of this element to solubilize in a solution of ammonium acetate, for which a soil sample of 5 or 10 g was used, selecting the amount of K present in the sample, choosing 5 g when it was higher than 500 ppm.

The active limestone content was determined using the Bernard calcimeter technique, comparing the volume of carbon dioxide released by a 5 g soil sample; and pure calcium carbonate, 0.1 g, diluted in 250 mL of ammonium oxalate, respectively. The amount of carbonate was also determined by the aforementioned technique, using in this case a 2.5 g sample of previously crushed soil.

The pH and electrical conductivity (E.C) of the soil were determined through the saturated paste using a pH meter and a conductivity meter, respectively. The saturated paste was prepared from a 250 g soil sample and 100 mL of distilled water.

The texture of the samples (amount of sand, silt, and clay) was obtained using the improved bouyoucos protocol from a 40 g soil sample which was sieved until it was composed of 2 mm diameter particles, subsequently following the USDA soil classification for particle size. Finally, the hydraulic conductivity of the soil at saturation (K_h_) was estimated from the model proposed by Saxton and Rawls [56] that used the values offered by the texture of the samples and their SOM content in its calculations.

#### 2.3.4. Evaluation of Seedling Growth in a Controlled Environment Chamber

##### Definition and Plant Material

A pot experiment was conducted to evaluate the impact of the soil on the growth of horticultural plants. The main purpose was to determine if the growth variables evaluated were related to the physical, chemical, and microbiological variables of the soil, depending on treatment. The horticultural species used were cucumbers (*Cucumis sativus cv.* Marketmore 76; Ramiro Arnedo S.A, Calahorra, Spain) and tomatoes (*Solanum lycopersicum* L. *cv.* Rio Grande; Ramiro Arnedo S.A., Calahorra, Spain). The methodology described by Marín-Guirao et al. [25] was used. Due to sample conservation problems, the evaluations were not performed with the soil sampled at the end of cultivation in the third year of the study.

##### Description of the Experiments

The trials were carried out in a controlled environment culture chamber with a photoperiod of 14 hours of light per day using low-pressure mercury vapor lamps and a luminous flux of 12,000 lm and temperature ranging between 21 °C and 25 °C. Each plant species was planted independently in 200 cm^3^ cylindrical pots (experimental unit) at the rate of 1 seed per pot. The pots contained the soil to be studied mixed with vermiculite in a 2 (soil):1 (vermiculite) *v*/*v* ratio and 5 replicates of each soil were made for each vegetable species. Seeds were previously disinfected with a 20% solution of commercial sodium hypochlorite (40 g·L^−1^) for 15 min and then rinsed with water. The trials lasted 30 days during which irrigation was applied on demand without using any fertilizer. The trials with the different soils (i.e., test, IF and PD) were repeated twice over time for each sampling.

##### Variables Analyzed and Measurement Process

The five variables that based on previous studies [24,25] were found to be the most representative and constant were evaluated at the end of the trials. These variables included the number of leaves, seedling height, root dry weight, aerial dry weight, and leaf area. Each cucumber and tomato seedling had any dirt removed by careful cleaning with water and was then fragmented into two portions: aboveground and belowground. They were then placed on filter paper and then put in a J.P-Selecta Dry-Big 2003720 oven (Barcelona, Spain) for 48 hours at a constant temperature of 72 °C to determine the dry weight using a Metter Toledo PB 303-S balance (Columbus, OH, USA) with a sensitivity of 0.001. Leaf area was determined using the free software ImageJ 1.48 (NIH Imagen, Bethesda, Maryland) after scanning leaves and leaflets with an Epson Perfection 1240 optical reader (Epson, Suwa, Japan). The number of leaves was quantified at the beginning of the process.

#### 2.3.5. Statistical Analysis

An analysis of variance (one-way ANOVA) was applied to compare the effect caused by the treatments applied to the soil (i.e., test, IF, and PD) for each of the variables analyzed (i.e., tomato fruit production and quality parameters, plant vigor parameters in a controlled environment chamber, and microbiological parameters). Previously, the assumptions of normality and homoscedasticity were tested using the Shapiro–Wilk and Bartlett tests, respectively. Likewise, Student’s *t*-test was used in cases where only two factors were compared (i.e., physical, and chemical parameters). The data were transformed in those cases where the requirements of the parametric test were not met. Kruskal–Wallis nonparametric test was used in cases where the data transformation was not sufficient to meet the assumptions. Tukey’s HDS post hoc test (in the parametric tests) and a pairwise comparison (nonparametric tests) was then applied to perform a pairwise comparison between the means and medians of the treatments, respectively, at a 95% confidence level. ANOVA and Tukey’s HDS tests were performed with the statistical package STATGRAPHIC CENTURION XVIII (Manugistic Incorporate, Rockville, MD, USA) for Windows while nonparametric tests (Kruskal–Wallis) were performed using Statistix v. 9.0.5 software (Analytical Software, Tallahassee, FL, USA).

Different stepwise linear regression models were calculated to determine the most relevant variables in the prediction of the plant vigor parameters evaluated (dependent variables) in the controlled environment chamber trials. The independent variables (i.e., predictor variables) considered were all the physical and chemical parameters determined in this study. The adjusted R^2^ value of each model was calculated to observe the reliability of the prediction. In addition, the statistical importance and significance of each predictor variable were determined through the adjusted and unadjusted beta coefficients and the t-test, respectively. The necessary conditions for the application of the stepwise regression model were assessed visually through the residual plots [57]. In this case, data processing was performed through the SPSS v. 26 statistical package (IBM, Armonk, NY, USA).

Fungal community data (at the genus scale) were compared among treatments (based on crop nutrition) using permutational multivariate analysis of variance (PERMANOVA) at the end of each year’s crop. PERMANOVA is a statistical test that calculates distance matrices between sources of variation to perform permutation tests for univariate or multivariate analysis of variance. This test calculates pseudo-F to obtain *p*-values [58]. For comparisons between treatments, pairwise PERMANOVA tests were performed from Monte Carlo simulation since there were few permutations possible to obtain an accurate *p*-value for inferences at an appropriately small significance level [59].

## 3. Results

### 3.1. Crop Production and Quality

**Figure 1:**
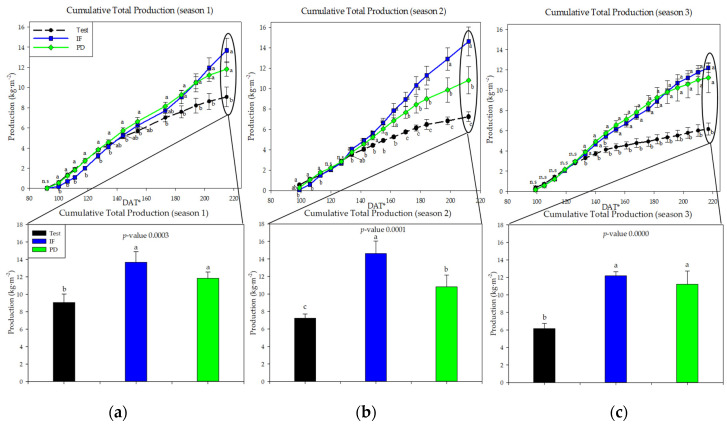
Cumulative tomato production in the three years of study (September–April cycles) as a function of crop nutrition: (**a**) Crop 1; (**b**): Crop 2; (**c**): Crop 3. Inorganic fertilization (IF); tomato plant debris (PD); no fertilization (test). Values (mean ± standard deviation). Different letters indicate significant differences (*p* ≤ 0.05, Tukey’s HDS test). DAT: days after transplanting.

Fertilization plans applied on the experimental plots (test, IF, and PD) significantly influenced the cumulative commercial production of tomato plants (Figure 1). In the three years studied, the production obtained from the plots that did not receive any fertilizer (test) was lower than that obtained from the plots that did receive fertilizer. Likewise, in Years 1 and 3 no differences were observed between PD and IF, but there were differences in Year 2 from 170 DAT and after in favor of the treatment with inorganic fertilizer (IF). The interpretation of the behavior of the accumulated production at the end of the second crop must be made taking into account that there was a *Botrytis cinerea* epidemic that was impossible to control due to environmental conditions (temperature and relative humidity). The mycosis began to produce symptoms and to cause production losses from 100 and 170 DAT, respectively. This epidemic did not occur neither in the first nor in the third campaign (mortality of the disease in the first and third campaigns was 0.0–1.5%, while in the second campaign it was 21.4 ± 10.34% for test, 41.1 ± 25.1% for IF, and 33.4 ± 4.4% for PD). A decrease in production was observed for all treatments between the first and third cycle, being 34.98% for test, 17.34% for IF, and 9.26% for PD.

In general, average yield per harvest showed a behavior analogous to that observed for final cumulative yield (Table 2). The IF and PD treatments showed similar fruit weight and diameter beginning with the second season. Non-fertilization had a negative influence on both parameters starting from the first growing season. Similarly, the interpretation of the results of the second year of the trial should be made taking into account the *Botrytis cinerea* epidemic that affected the tomato plants during the second season. The fertilization plans (test, IF, and PD) did not seem to influence the parameters of firmness, soluble solids, and acidity of the fruits due to the expression of a variable or uniform response of the parameters during the production cycles.

### 3.2. Soil Microbiota

The study of soil microbiota was aimed at finding a relationship between the addition of tomato plant debris and its influence on crop productivity [25]. It was also intended to know whether the effect of solarization with or without organic amendment caused a degradation of the arable soil microbiota [60].

#### 3.2.1. Total Population (Bacteria and Fungi)

**Figure 2:**
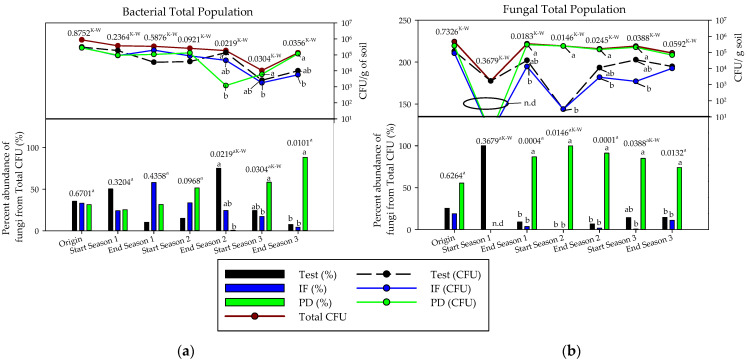
Soil microbiota in the three years of study (September–April cycles) as a function of crop nutrition: (**a**): total bacterial population (CFU and percentage abundance); (**b**): total fungal population (CFU and percentage abundance). inorganic fertilization (IF; n = 4); tomato plant debris (PD; n = 4); no fertilization (Test; n = 4). N.d: non detected. Values (average). Different letters above the bars and in the evolution lines indicate significant differences. (*p* ≤ 0.05, Tukey’s HDS test), ^a^: $arcsen\left(\sqrt{x}\right)$; ^K-W^: Kruskal–Wallis test).

Figure 2 enables a reading of the results that are repeated in the three years of experimentation. Solarization with or without tomato plant debris significantly decreases the density of culturable bacteria and fungi. At the end of each analysis campaign a significant increase in soil microbiota is observed, and this recovery is more evident in those plots where tomato plant debris were added.

The reiterated addition of tomato plant debris (PD) during three consecutive years modified the population of culturable soil bacteria, significantly increasing their relative abundance compared to inorganic fertilization (IF) and no fertilization (test), which registered a decrease throughout the experiment (Figure 2). At the end of the trial, the density of bacteria in the plots that received the PD treatment showed the same order of magnitude as at the beginning of the study (10^5^ UFC/g of soil). The test decreased by one order of magnitude and IF by two orders of magnitude.

General speaking, soil solarization treatments had a depressive effect on fungal populations. This effect was more evident during the first year of the trial where no fungal CFU was detected in the soil of the PD and IF plots by the analytical technique used (Figure 2). However, in the second and third years this effect was not so evident in the plots that received tomato plant debris, although it was detected in the soil of the test and IF plots. The fungal population of PD surpassed test and IF at some points by one or two orders of magnitude, although at the end of the trial all treatments reduced their concentration by one order of magnitude. The PD treatment reached a higher relative abundance of filamentous fungi than IF from the end of the first trial.

**Figure 3:**
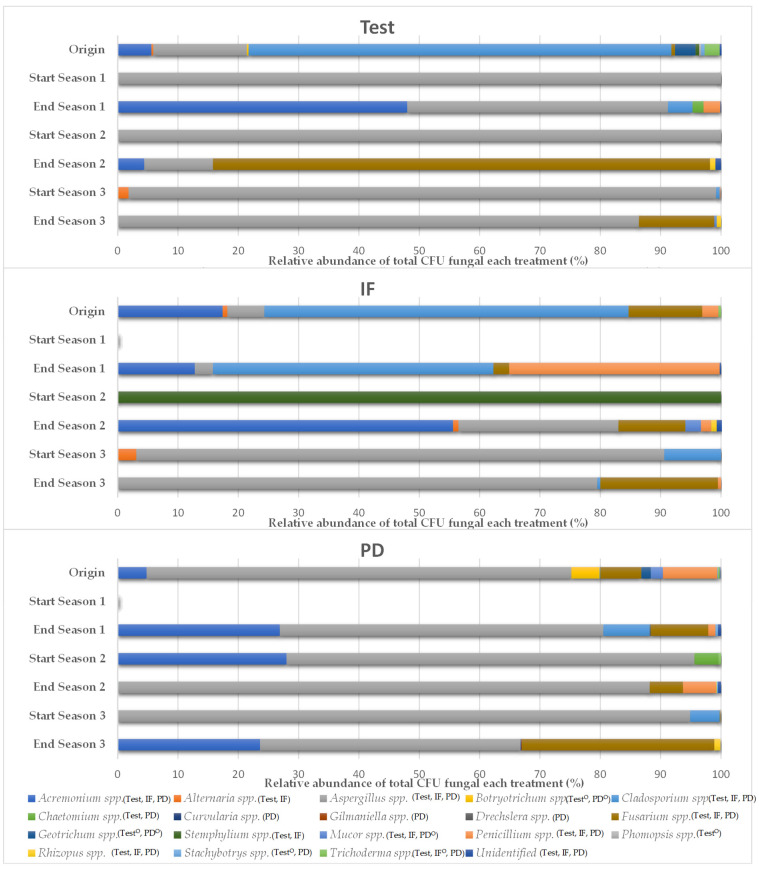
Relative abundance of fungal genera in the three years of study (September–April cycles) as a function of crop nutrition. Inorganic fertilization (IF; n = 4); tomato plant debris (PD; n = 4); no fertilization (test; n = 4). Values (average). ^O^: isolated only in the initial sampling.

Nineteen different fungal genera were identified by morphology during the trial, three of which (*Botryotrichum* spp., *Geotrichum* spp. y *Phomopsis* spp.) were only isolated in the initial analysis before the first solarization treatment. Only *Aspergillus* spp. and *Cladosporium* spp. tended to be isolated in all treatments after solarization (Figure 3). At the end of cultivation this number increased to six (*Acremonium* spp., *Aspergillus* spp., *Cladosporium* spp., *Fusarium* spp., *Penicillium* spp., and *Rhizopus* spp.). Three of them (*Acremonium* spp., *Aspergillus* spp. *Fusarium* spp.) were found in the plots that received tomato plant debris at all sampled times, which did not occur in the plots without fertilization or with inorganic fertilization. After the first solarization treatment, and considering the rest of the trial, the plots with tomato plant debris presented the highest number of filamentous fungi (13 genera). In general, *Aspergillus* spp. was the fungal genus that reached the highest expression at all times of analysis and treatments while accounting for an average of 61.4% of the CFU/g of soil.

#### 3.2.2. Descriptive Variables of the Fungal Community

None of the descriptive variables of the fungal community showed differences among the different treatments (test, PD, and IF) during the three years of this study (Table 3). In general, the descriptive parameters of the fungal community showed a decrease for all treatments at the end of the trial compared to those observed at the beginning of the experiment.

PERMANOVA analysis revealed differences in the composition of the cultivable fungal community as a function of the treatments applied (test, IF, and PD) at the end of the cultivation of the first two years of the trial (Table S1) where the experimental plots that received tomato plant debris (PD) showed a different fungal composition than inorganic fertilization (IF) and no fertilization (Test).

#### 3.2.3. Fusarium Fungi

**Figure 4:**
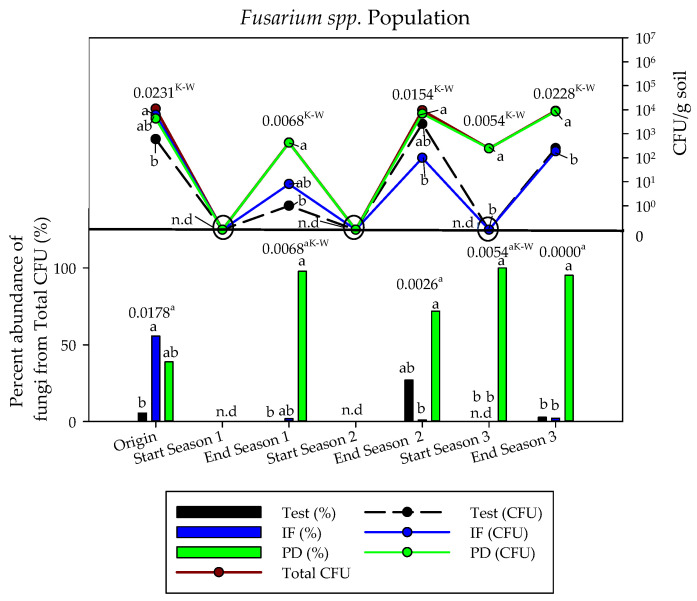
Soil *Fusarium* microbiota in the three years of study (September–April cycles) as a function of crop nutrition. Inorganic fertilization (IF; n = 4); tomato plant debris (PD; n = 4); no fertilization (Test; n = 4). N.d: not detected. Values (average). Different letters above the bars and in the evolution lines indicate significant differences. (*p* ≤ 0.05, Tukey’s HDS test), ^a^: $arcsen\left(\sqrt{x}\right)$; ^K-W^: test Kruskal–Wallis test.

Figure 4 shows the results regarding culturable filamentous fungi of the genus *Fusarium* during the three campaigns. These results corroborate the findings for the general cultivable bacterial and fungal microbiota.

Generally speaking, soil solarization performed before the start of cultivation had a depressive effect on filamentous fungi of the genus *Fusarium* for the three treatments, which made them undetectable in the soil by the analytical technique used, regardless of the treatment (Figure 4). In the third year of the study, the soil that had received tomato plant debris showed values close to the initial ones. In any case, a tendency to reconstitute was detected at the end of the crop, especially in the soil that received tomato plant debris. Thus, at the end of the trial, the population density was higher than those of the other treatments with values close to the initial ones.

**Figure 5:**
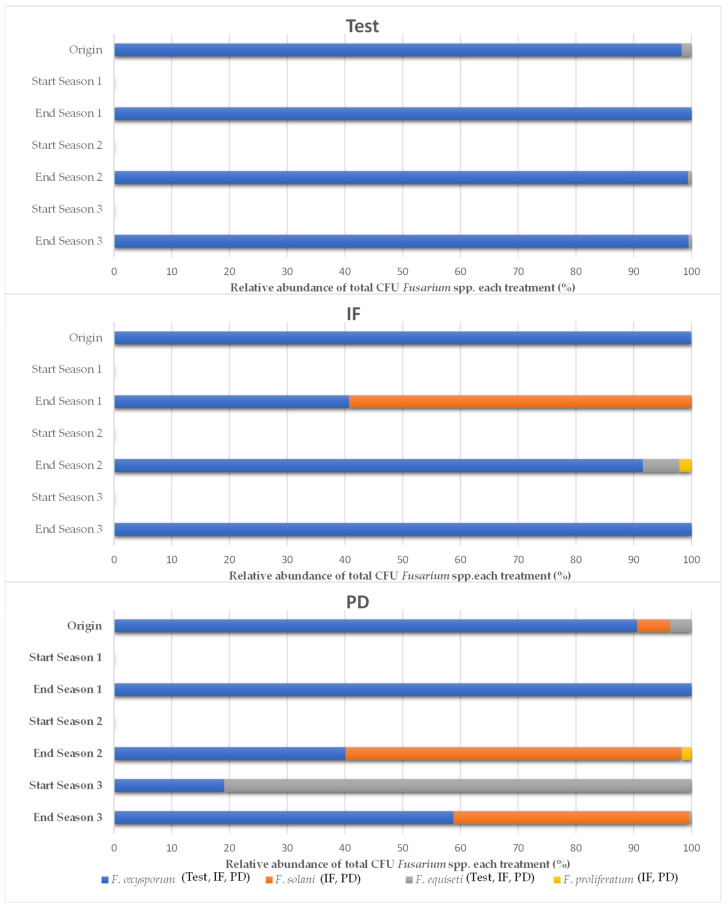
Relative abundance of *Fusarium* species in the three years of study (September–April cycles) as a function of crop nutrition. Inorganic fertilization (IF; n = 4); tomato plant debris (PD; n = 4); no fertilization (test; n = 4). Values (average).

A total of four different species belonging to the genus *Fusarium* (*F. oxysporum*, *F. solani*, *F. equiseti*, and *F. proliferatum*) were identified by their morphology (Figure 5). Only *F. oxysporum* and *F. equiseti* species were identified in the soil of the plots that did not receive fertilization (test), while in the soil with inorganic fertilization (IF) and with tomato plant debris (PD) *F. solani* and *F. proliferatum* were also isolated. In general, when *Fusarium* spp. were present, *F. oxysporum* was always the species with the highest expression, accounting for 74.9% of the total CFU counted.

### 3.3. Physical and Chemical Variables of Soil Samples

**Figure 6:**
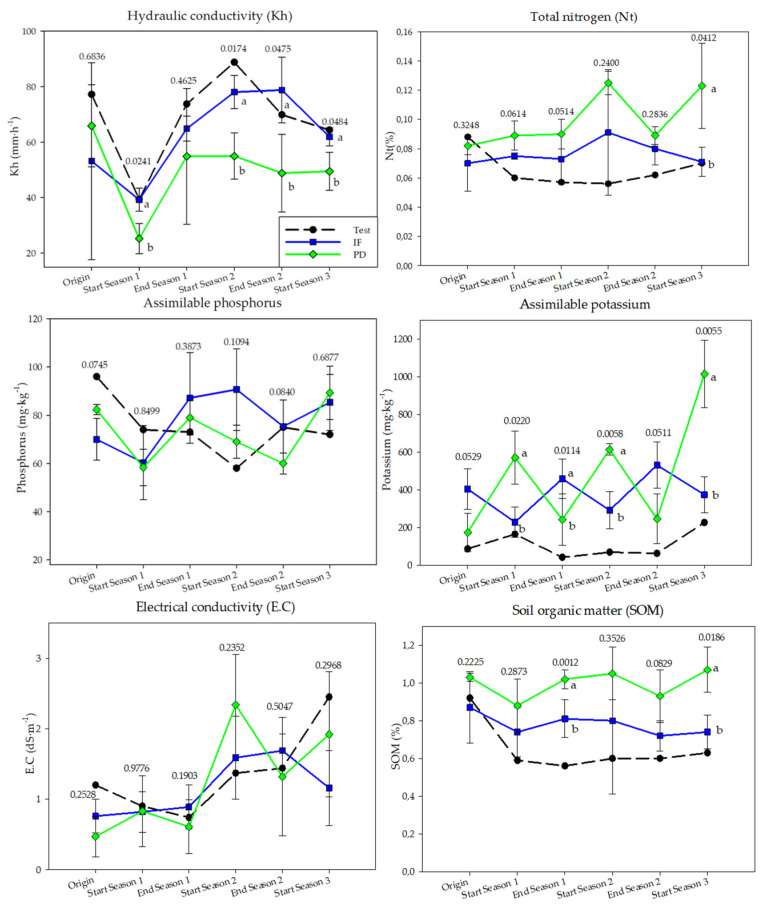
Soil physicochemical parameters in the three years of study (September–April cycles) as a function of crop nutrition. Inorganic fertilization (IF; n = 3); tomato plant debris (PD; n = 3); no fertilization (test; n = 1). Values (mean ± standard deviation). Different letters indicate significant differences between IF and PD (*p* ≤ 0.05, Student’s *t*-test).

The sand and clay contents maintained a constant trend during the trial with no influence of the treatment applied on these parameters (Figure S1). The addition of tomato plant debris increased the silt content and decreased the hydraulic conductivity of the soil compared to inorganic fertilization (Figure 6).

Carbonate, assimilable phosphorus, C/N ratio, E.C., and limestone contents were not influenced by the fertilization plan applied during the experiment (Figure 6 and Figure S1). However, each of the treatments increased the E.C. of the soil. In addition, the experimental plots where tomato plant debris was applied showed the highest limestone concentration and an increase in the C/N ratio was observed after solarization. Soil pH was significantly increased by the addition of tomato plant debris compared to inorganic fertilization. At the end of the trial, SOM values were higher in soil that received vegetables during the three years of study while detecting an increase of 3.8% with respect to the initial value, while IF registered a decrease of 14.9%.

In the three years of the study, assimilable potassium values increased after the addition of tomato plant debris and solarization, while they decreased at the end of the crop (Figure 6). In the case of the IF treatment, the trend is the opposite. At the beginning of the crop, the assimilable potassium content was always higher in the plots that received tomato plant debris compared to the rest of the treatments.

### 3.4. Plant Growth in a Controlled Environmental Chamber

**Figure 7:**
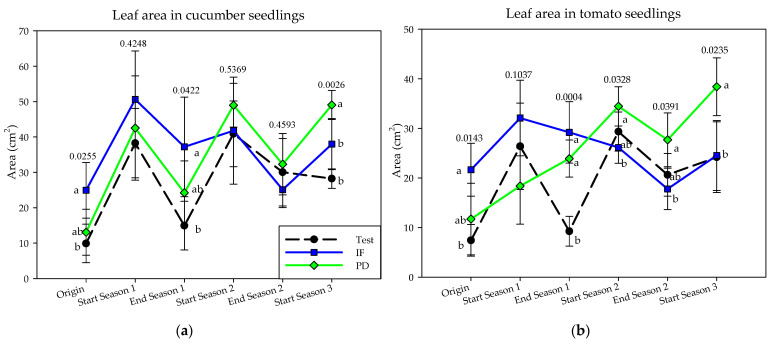
Leaf area of seedlings grown in controlled chamber conditions in the three years of study (September–April cycles) as a function of crop nutrition: (**a**): cucumber; (**b**): tomato. Inorganic fertilization (IF; n = 4); tomato plant debris (PD; n = 4); no fertilization (Test; n = 4). Values (mean ± standard deviation). Different letters in the evolution lines indicate significant differences (*p* ≤ 0.05, Tukey’s HDS test).

This research aimed to evaluate the modifications found in solarized soil with or without tomato plant debris. The model used enabled the evaluation of vigor expression in tomato and cucumber seedlings. The tests suggest that the addition of plant debris with solarization produced a greater expression of seedling vigor (number of leaves, height, aerial dry weight, root dry weight and leaf area) (Figure 7 and Figures S2 and S3). This expression was most visible in the leaf area, thus corroborating the results obtained in the greenhouse.

#### 3.4.1. Tomato

The application of the three treatments did not show a homogeneous behavior among the plant vigor variables measured (Figure 7 and Figure S2). However, the repeated addition of tomato plant debris progressively increased the leaf area of tomato seedlings, while inorganic fertilization showed an inverse trend. At the end of the trial, the PD treatment showed a statistically higher aerial dry weight and leaf area than test and IF. Root dry weight increased at the end of each year’s production cycle in all three treatments.

#### 3.4.2. Cucumber

The vigor variables measured on cucumber seedlings showed a lower sensitivity to the effect of the applied treatments (Figure 7 and Figure S3). At the end of the trial, the addition of tomato plant debris statistically differentiated the leaf area of cucumber seedlings compared to inorganic fertilization and no fertilization. The leaf area of cucumber seedlings increased on average after solarization for all three treatments regardless of whether or not tomato plant debris was applied. Non-fertilization had a negative influence on the seedling height at the end of the experiment.

The stepwise linear regression analysis showed a variable response to the prediction of the independent variables (vigor parameters) of both vegetable species (tomato and cucumber) (R^2^: 0.106–0.343) (Table S2). However, the predictive models coincided in reporting C/N ratio and carbonate concentration in both vegetable species as factors that manifested a direct and inversely proportional relationship in the prognosis of some of the vigor parameters (number of leaves, height, root dry weight, and leaf area).

## 4. Discussion

This research, which was carried out over three years, aimed to evaluate the effects of the repeated supply of tomato plant debris from the previous season versus the use of inorganic cover fertilization and no fertilization on tomato production and crop quality; physical, chemical, and biological soil variables that determine soil fertility, and on the vigor of tomato and cucumber seedlings grown under controlled conditions. In all cases, the solarization technique was applied during the summer months before the start of cultivation, and production cycles were of 215 DAT. Previous research concluded that the addition of tomato plant debris was sufficient for the correct development of a greenhouse tomato crop when production cycles were lower than 170 DAT. This achieved the same yield as when applying a conventional inorganic fertilizer while also maintaining the main organoleptic properties of the fruit [20,21]. However, the aforementioned study included two crop cycles and it did not report information on the effects on soil parameters or the evaluations in a controlled environment chamber using bioassays that help to better interpret the effects on these soil parameters which determine its fertility. In addition, greater precision has been achieved concerning the analytical findings. The results of the three years of testing in the present study suggest that exclusive fertilization with tomato plant debris produces a yield and crop quality similar to that obtained with traditional inorganic manure in production cycles of 215 DAT. Several authors have reported a similar result when they analyzed the production of tomato crops with only organic fertilizer (compost, bone meal, blood or hoof meal, chicken, sheep or turkey manure, and plant debris) versus conventional fertilization, both with or without using pre-transplant solarization and conventional fertilization [61,62,63,64,65,66] to obtain a tomato fruit of similar quality [62,63,64]. Some investigations have also reported decreases in the production of a bell pepper crop nourished with tomato plant debris and compost compared to the conventional crop. It should be noted that a small amount of inorganic fertilizer was added to the organic fertilization and that the solarization technique was not used [22]. Thus, the technique of soil solarization combined with organic amendments, also known as biosolarization, has resulted in increases in the production of different crops. Nonetheless, the effects reported in these studies have been mostly the result of the control of pathogens that limit the correct development of the plants and in crops that have incorporated inorganic nutrition [35,36,37,38,67]. It should also be noted that the soil biosolarization technique can have an influence on soil fertility [28,29,30,31,32,68] in conjunction with the control of soil pathogens. The use of the biodisinfection technique seems to favor the decomposition of organic amendments, in our case of plant debris from the previous crop. The application of this technique could help to decrease the time necessary for the decomposition of the material. This would provide the plants with a higher content of nutrients needed for growth in a shorter time [29,32]. In addition, the use of the solarization technique helps to limit pests and diseases that may be associated with plant debris incorporated into the soil [20,69]. The presence of these organisms in combination with plant material is normal after long-term production cycles, and it is essential to avoid their expression during the following production cycle in order not to limit crop production. The repetition of non-fertilization resulted in a continuous decrease in final yield similar to what occurred in other investigations [61]. However, some authors did not obtain differentiated production between their treatments fertilized only with organic amendments and the absence of fertilization [69].

In our trial, the study of the bacterial, fungal, and culturable fusarium microbiota showed a depressive effect after applying solarization. At the end of each campaign this microbial fraction was able to reestablish itself while becoming more evident in the experimental plots where tomato plant debris were added, which showed higher values than in the other treatments. Other investigations that have evaluated the depressive effect of greenhouse solarization on the microbiota of the arable soil have reported this capacity of the microbial population to recover at the end of the production cycle [60,70,71], although in some, there was no repetition of the solarization technique over time [60]. On the other hand, the classical biodiversity parameters of the fungal community were similar among the fertilization plans applied. However, repeated solarization caused a decrease in the values obtained, which has also been observed in the research conducted by Marín-Guirao et al. [60]. Our research suggests that the addition of tomato plant debris may have modified the composition of the filamentous fungi fraction during the first two years of the trial. Accordingly, other investigations have observed a change in the fungal community composition of a maize crop by incorporating straw versus the conventional crop [72]. The total number of fungal genera isolated from the greenhouse soil, considering all treatments and samplings, was 19, those being *Acremonium* spp., *Alternaria* spp., *Aspergillus* spp., *Cladosporium* spp., *Fusarium* spp., *Penicillium* spp., and *Rhizopus* spp. the most frequently isolated. This is similar to the findings of other studies that have used the technique of successive dilutions to study the fungal microbiota associated with horticultural greenhouses [24,60,70] and rainfed almond soil [41]. In the analyses for the genus *Fusarium there were* four different species isolated, the most abundant being *F. oxysporum*. Some experiments carried out in greenhouse cultivation report this species as the most abundant in the analyses performed after the end of cultivation, with *F*. *solani* being the most dominant after applying soil disinfection [71]. Likewise, a dominance of *F. oxysporum* has also been observed in soils where asparagus is grown outdoors, although the expression of these species is not homogeneous in all asparagus fields where the dominance of *F. equiseti* also stands out [73]. Thus, various functionalities are attributed to the isolated fungal microbiota, although in this research an independent study was not carried out to verify them. Different studies have observed the ability of fungal organisms to solubilize phosphorus (*Alternaria* spp., *Aspergillus* spp., *Penicillium* spp., *Trichoderma* spp., *Rhizopus* spp.) [74,75,76], participate in nitrification processes (*Aspergillus* spp., *Penicillium* spp.) [77], promote plant growth (*Trichoderma* spp.) [78] or practice saprophytism (*Aspergillus* spp., *Penicillium* spp., *Trichoderma* spp.) [79,80]. However, these effects are usually not fully clarified when considering the soil environment as a whole where multiple factors can condition the behavior of these microorganisms so that the simple modification of one of these conditions can determine the microoganisms present in the soil [78].

In turn, the results of this research suggest that the addition of tomato plant debris with solarization increased the total nitrogen and assimilable potassium content. Mauromicale et al. [30,31] and Nuñez-Zofio et al. [29] observed an increase in these soil variables after applying their solarization protocols with organic amendments, while Seo et al. [68] only reported an increase in nitrate content. These authors observed an increase in assimilable phosphorus and soil electrical conductivity, something that did not occur in our trial possibly due to the difference in origin and nutrient composition of the organic amendments used. Likewise, the results suggest that the organic matter content of the soil did not increase significantly with the addition of tomato plant debris for three consecutive years. Other authors who have used solarization with different organic amendments did observe a significant increase in this soil variable throughout their experiments [24,29,81]. Thus, the higher content of some nutrients could have helped to maintain the final production of the plots that received tomato plant debris at levels similar to those obtained in the plots with inorganic mulch fertigation. The results suggest an improvement in soil hydraulic conductivity in the plots where solarization with tomato plant debris was applied. Biosolarization is a technique capable of modifying the soil infiltration rate as a consequence of the incorporation of organic amendments and their impact on soil structure [28]. This modification of soil hydraulic conductivity could have direct implications on the dynamics of irrigation applied to tomato crops (frequency and allocation), thus improving the water footprint of this production system compared to conventional fertilization.

The addition of tomato plant debris through solarization improved the vigor variables of the seedlings grown in a controlled environment chamber, mainly their leaf area and the dry weight of the aerial part, which are the parameters that best determine the vigor of the seedlings. The results obtained in this model support the findings obtained under greenhouse conditions. Thus, the leaf area of the different treatments increased after the application of solarization indistinct of the addition of tomato plant debris. Marín-Guirao et al. [24] obtained an increase in the vigor of their seedlings after applying a solarization protocol with organic amendments in a greenhouse where a commercial cucumber crop was grown. Similarly, the addition of organic amendments in a rainfed almond crop increased the vigor variables of cucumber seedlings compared to the conventional crop [41,82]. Our experimentation illustrates a low correlation obtained between the physicochemical variables of the soil and the vigor of cucumber seedlings, especially in the case of C/N ratio being the soil variable with the highest interdependence. Other studies have obtained a high correlation between soil productivity and physical, chemical, and microbiological variables, even postulating SOM as the most relevant variable in soil fertility, which in turn had a high correlation with fungal density and diversity. All of this is applicable when considering greenhouse soil with cucumber or tomato monocultures that showed a great disparity in their SOM content [25]. Although no relationships were found, the soil microbiota could have influenced these results. In our trial, a decreasing evolution of leaf area was observed in the treatment that only received inorganic fertilizers. Usero et al. [83] observed a negative influence on root dry weight, aerial dry weight and leaf area of tomato seedlings grown in pots under greenhouse that had been treated with an inoculum prepared from the microbiota present in the soil of a commercial greenhouse fertilized only with synthetic inorganic fertilizers versus others fertilized with organic amendments and a treatment without inoculation. This model with seedlings grown in a controlled conditions chamber allowed us to explain the possible influence of tomato plant debris on soil fertility, expressed by its vigor. This relationship could not be established with the physicochemical analyses performed on the soils studied (test, IF and PD). Their analytical performance remained constant in several of the parameters measured or did not show a clear difference, a behavior similar to that observed in other studies [24,25,41]. The suggested improvement in soil fertility observed through the leaf area of the seedlings could have influenced in keeping the yield of the tomato crop fertilized with only tomato plant remains from the previous crop similar to that offered by the conventional crop with inorganic fertilization.

## 5. Conclusions

The repeated reuse of tomato plant debris obtained at the end of the crop cycle as an organic amendment has a positive effect on the physical, chemical, and biological parameters that determine the fertility of greenhouse soil. Thus, by incorporating this material into the soil, the needs of the tomato crop are satisfied in cycles with a duration of approximately 215 DDT in reaching yields equal to those obtained by means of exclusive fertilization with conventional inorganic synthesis fertilizers while also maintaining the organoleptic quality of the fruit. The reuse by the producer of this vegetable by-product solves the problems linked to the external management of the material and contributes to a reduction in production costs in intensive horticultural farms through a more sustainable agricultural practice in accordance with the principles of the circular economy. Future studies should focus on the reuse of plant material from other horticultural species to determine its suitability for reuse as an organic amendment with benefits for crops and the sustainability of the greenhouse horticultural production process.

## Figures and Tables

**Table 1:** Nutrient sources used in the different experimental plots.

Nutrient Source	Composition
Irrigation water (test, IF, and PD)	E.C: 0.48 ± 0.03 dS·m^−1^; NO_3_^−^: 0.04 ± 0.00 mmol·L^−1^; H_2_PO_3_^−^: 0.03 ± 0.00 mmol·L^−1^; K^+^: 0.08 ± 0.01 mmol·L^−1^; SO_4_^2−^: 0.07 ± 0.00 mmol·L^−1^; Ca^2+^: 0.34 ± 0.01 mmol·L^−1^; Mg^2+^: 0.19 ± 0.00 mmol·L^−1^; HCO_3−_: 0.89 ± 0.12 mmol·L^−1^; CO_3_^2−^: 0.40 ± 0.00 mmol·L^−1^; Cl^−^: 3.36 ± 0.40 mmol·L^−1^; Na^+^: 2.98 ± 0.58 mmol·L^−1^.
Nutrient solution (IF)	CE: 3.00 dS·m^−1^; NO_3_^-^: 18 mmol·L^−1^; H_2_PO_3_^−^: 1.5 mmol·L^−1^; K^+^: 10.5 mmol·L^−1^; SO_4_^2−^: 5.25 mmol·L^−1^; Ca^2+^: 6.75 mmol·L^−1^; Mg^2+^: 3 mmol·L^−1^.
Tomato plant debris (PD)	OM: 51.8%; N: 1.86%; P: 2.69%; K: 8.94%; Mg: 1.31%; Ca: 6.41%; Na: 1.60%.

E.C: electrical conductivity; OM: organic matter.

**Table 2:** Tomato fruit yield and quality parameters in the three years of study (September–April cycles) as a function of crop nutrition. Values (mean ± standard deviation).

		Mean Yield (kg·m^−2^)	Fruit Weight (g)	Size (mm)	Firmness (kg·m^−2^)	Soluble Solids (◦Brix)	Fruit Acidity (pH)
Season 1	Test (n = 4)	0.65 ± 0.07 b	108.20 ± 4.17 b	58.36 ± 0.40 c	5.15 ± 0.28 a	5.46 ± 0.14 a	3.91 ± 0.04 b
	IF (n = 4)	0.98 ± 0.09 a	125.18 ± 11.05 a	63.53 ± 0.79 a	4.50 ± 0.19 b	5.01 ± 0.08 b	4.03 ± 0.03 a
	PD (n = 4)	0.85 ± 0.05 a	115.82 ± 4.33 ab	60.84 ± 1.00 b	4.56 ± 0.18 b	5.47 ± 0.11 a	4.01 ± 0.03 a
	*p*-value	0.0003	0.0276	0.0000	0.0043	0.0003	0.0012
Season 2	Test (n = 4)	0.48 ± 0.03 c	99.06 ± 4.87 b	56.59 ± 1.67 b	5.08 ± 0.20 ab	5.50 ± 0.21	4.20 ± 0.02
	IF (n = 4)	0.97 ± 0.10 a	126.59 ± 3.87 a	62.66 ± 0.56 a	4.84 ± 0.16 b	5.28 ± 0.19	4.23 ± 0.05
	PD (n = 4)	0.72 ± 0.09 b	114.70 ± 11.05 a	60.29 ± 2.52 a	5.38 ± 0.20 a	5.53 ± 0.36	4.17 ± 0.06
	*p*-value	0.0000	0.0016	0.0030	0.0104	0.3788	0.2417
Season 3	Test (n = 4)	0.34 ± 0.03 b	72.10 ± 4.10 b	50.18 ± 1.13 b	5.33 ± 0.21 a	5.64 ± 0.11	3.85 ± 0.03
	IF (n = 4)	0.68 ± 0.02 a	104.70 ± 4.80 a	59.18 ± 1.04 a	4.78 ± 0.88 ab	5.54 ± 0.15	3.85 ± 0.02
	PD (n = 4)	0.62 ± 0.08 a	95.60 ± 10.80 a	56.71 ± 2.43 a	4.00 ± 0.41 b	5.58 ± 0.25	3.89 ± 0.03
	*p*-value	0.0000	0.0003	0.0001	0.0285	0.7636	0.1588

Inorganic fertilization (IF); tomato plant debris (PD); no fertilization (test). Different letters between columns and seasons indicate significant differences. (*p* ≤ 0.05, Tukey’s HDS test).

**Table 3:** Biodiversity indices of the fungal community in the three years of study (September–April cycles) as a function of crop nutrition. Values (mean ± standard deviation).

Sampling	Treatments	Simpson’s Diversity Index	Shannon–Wiener’s Diversity	Margalef’s Index	Pielou’s Equity Index	Nº of Genera
Origin	Test (n = 4)	0.52 ± 0.18	1.07 ± 0.45	0.45 ± 0.14	0.59 ± 0.20	6.00 ± 1.00
	IF (n = 4)	0.45 ± 0.13	0.89 ± 0.28	0.33 ± 0.11	0.57 ± 0.09	4.67 ± 1.15
	PD (n = 4)	0.52 ± 0.24	1.12 ± 0.52	0.48 ± 0.14	0.59 ± 0.22	6.67 ± 2.08
	*p*-value	0.8810	0.7888	0.3619	0.9910	0.3170
End Season 1	Test (n = 4)	0.43 ± 0.28	0.79 ± 0.47	0.30 ± 0.08	0.56 ± 0.33	4.00 ± 0.82
	IF (n = 4)	0.43 ± 0.25	0.77 ± 0.43	0.31 ± 0.14	0.57 ± 0.27	3.75 ± 1.50
	PD (n = 4)	0.45 ± 0.24	0.79 ± 0.42	0.34 ± 0.10	0.48 ± 0.21	5.25 ± 1.26
	*p*-value	0.9748 ^W^	0.9961	0.8370	0.8762	0.2326
End Season 2	Test (n = 4)	0.39 ± 0.27	0.65 ± 0.40	0.30 ± 0.17	0.57 ± 0.26	3.50 ± 1.29
	IF (n = 4)	0.34 ± 0.27	0.54 ± 0.37	0.28 ± 0,22	0.57 ± 0.36	3.25 ± 1.89
	PD (n = 4)	0.23 ± 0.18	0.43 ± 0.30	0.34 ± 0.08	0.26 ± 0.17	5.00 ± 0.82
	*p*-value	0.6609	0.6946	0.8924	0.2259	0.2174
End Season 3	Test (n = 4)	0.18 ± 0.30	0.31 ± 0.49	0.15 ± 0.13	0.38 ± 0.49	2.25 ± 0.96
	IF (n = 4)	0.06 ± 0.06	0.14 ± 0.13	0.11 ± 0.09	0.22 ± 0.06	2.00 ± 0.82
	PD (n = 4)	0.26 ± 0.14	0.46 ± 0.23	0.20 ± 0.08	0.38 ± 0.15	3.25 ± 0.96
	*p*-value	0.3141 ^w^	0.3413 ^w^	0.4687	0.4474 ^Z^	0.1724

Inorganic fertilization (IF); tomato plant debris (PD); no fertilization (Test). Nº of Genera: number of genera. Different letters between columns and sampling indicate significant differences. (*p* ≤ 0.05, Tukey’s HDS test), ^W^: $\frac{1}{log\left(x\right)}$, ^Z^:$\sqrt{x}$;).

## Supplementary Materials

The following are available online at https://www.mdpi.com/article/10.3390/ijerph182111544/s1, Figure S1: soil physicochemical parameters in the three years of study (September–April cycles) as a function of crop nutrition, Figure S2: number of leaves, height and root and aerial dry weight of cucumber seedlings grown in controlled chamber conditions in the three years of study (September–April cycles) as a function of crop nutrition, Figure S3: number of leaves, height and root and aerial dry weight of tomato seedlings grown in controlled chamber conditions in the three years of study (September–April cycles) as a function of crop nutrition, Table S1: PERMANOVA analysis of the fungal community in the three years of study (September–April cycles) as a function of crop nutrition, Table S2: stepwise linear regression models evaluating the prediction of growth variables of tomato and cucumber seedlings grown in controlled chamber conditions.
