# Supplementary material for: Effect of Repeated Plant Debris Reutilization as Organic Amendment on Greenhouse Soil Fertility
Source: Int J Environ Res Public Health. 2021 Nov 3;18(21):11544. doi: 10.3390/ijerph182111544 (PMC8583493; doi:10.3390/ijerph182111544)
Supplement: Supplementary file 1 [file ijerph-18-11544-s001.zip › ijerph-1442644-supplementary.pdf]

Table S1. PERMANOVA analysis of the fungal community in the three years of study (September-April cycles) as a function of crop nutrition.

Global test			Pairwise test		
**Sampling**	**Pseudo-F**	***p*-value**	**Treatments**	**t-statistic**	**P(MC)**
Origin	1.6251	0.1147	Test vs IF	1.1502	0.3077
			Test vs PD	1.3067	0.2116
			PD vs Test	1.3463	0.1976
End Season 1	2.552	0.0048	Test vs IF	1.1646	0.2644
			Test vs PD	1.7549	0.0435
			PD vs Test	1.8049	0.0289
End Season 2	4.1707	0.0045	Test vs IF	0.8631	0.5517
			Test vs PD	2.6264	0.0062
			PD vs Test	2.6269	0,0036
End Season 3	1.081	0.3865	Test vs IF	0.6530	0.6829
			Test vs PD	1.1025	0.3253
			PD vs Test	1.3161	0.1938

Inorganic fertilization (IF); tomato plant debris (PD); no fertilization (Test).

Table S2. Stepwise linear regression models evaluating the prediction of growth variables of tomato and cucumber seedlings grown in controlled chamber conditions.

	**Predicted variable**	**Adjusted R^2**	**s^2, df**	**Predictor variable**	**β**	**Partial t test, p-value**
Tomato	Nº of leaves	0.112	0.52541, 33	Constant	4.443±0.028	<0.001
				HCO_3^-	-0.064±0.028	0.028
	Height	0.202	0.98328, 33	Constant	1.820±1.321	0.177
				C/N	0.718±0.232	0.004
	Aerial dry weight[1]	-	-	-	-	-
	Roots dry weight	0.276	0.01865, 32	Constant	-0.024±0.027	0.365
				C/N	0.016±0.005	0.002
				Clay	-0.003±0.001	0.016
	Leaf area	0.108	8.25302, 33	Constant	15.128±5.100	0.006
				Nt	127.243±56.168	0.030
Cucumber	Nº of leaves	0.343	0.40582, 33	Constant	2.400+0.808	0.006
				HCO_3^-	-0.071+0.022	0.003
				SOM	0.843±0.357	0.025
				C/N	0.211±0.098	0.039
	Height	0.133	0.38085, 33	Constant	4.213±0.007	<0.001
				Silt	0.018±0.007	0.018
	Aerial dry weight[1]	-	-	-	-	-
	Roots dry weight	0.106	0.01761, 33	Constant	0.023±0.024	0.338
				C/N	0.009±0.004	0.031
	Leaf area	0.249	12.19180, 32	Constant	77.514±16.90	<0.001
				HCO_3^-	-1.815±0.642	0.008
				K+	0.019±0.008	0.022

[1]: Note that a model to predict roots dry weight of tomato seedlings could not be calculated, as none of the variables were selected during the regression

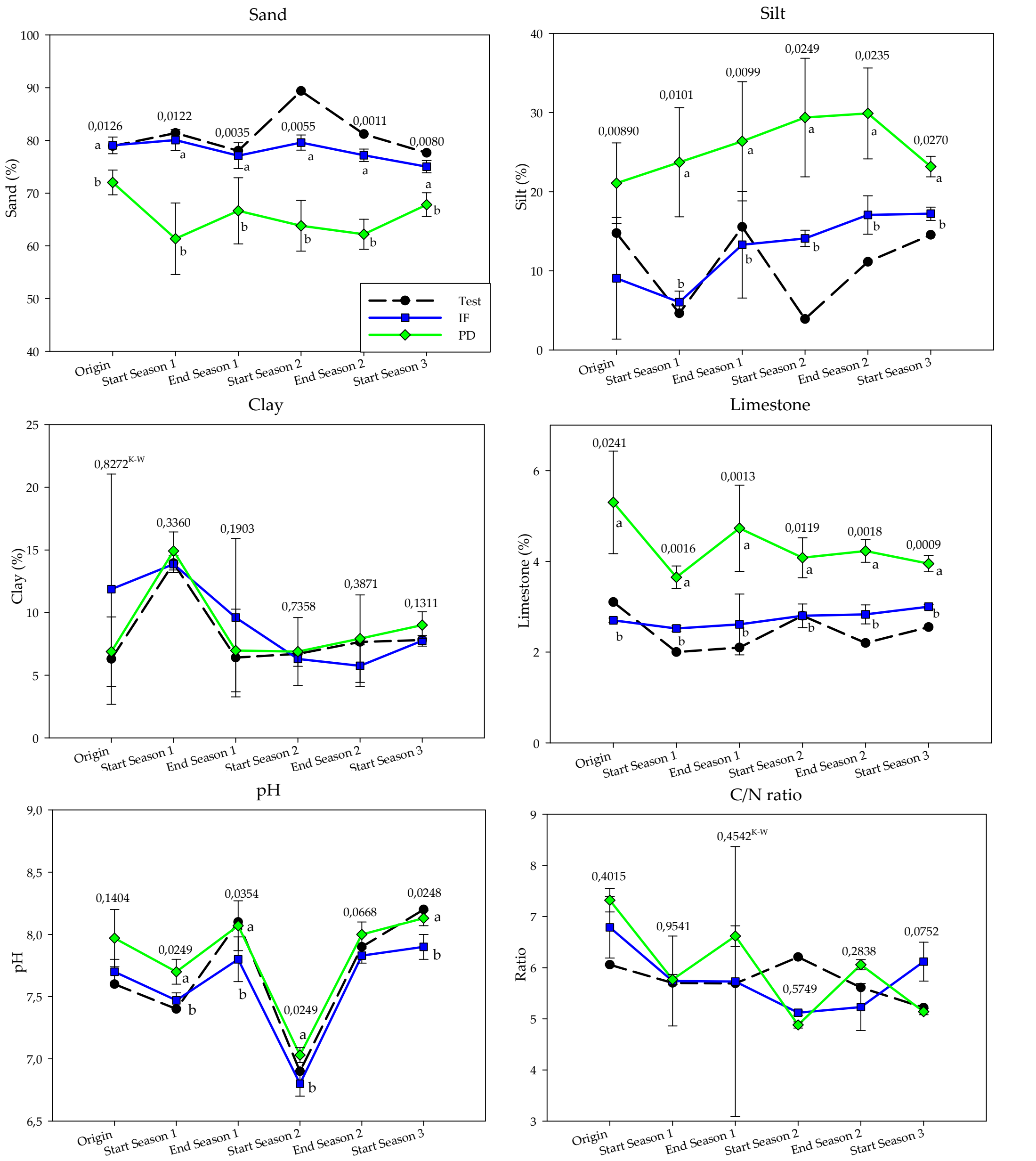

Figure S1. Soil physicochemical parameters in the three years of study (September-April cycles) as a function of crop nutrition. Inorganic fertilization (IF; n=3); tomato plant debris (PD; n=3); no fertilization (Test; n=1). Values (mean ± standard deviation). Different letters indicate significant differences between IF and PD ($p \leq 0.05$, Student's t-test; K-W: test Kruskal-Wallis test).

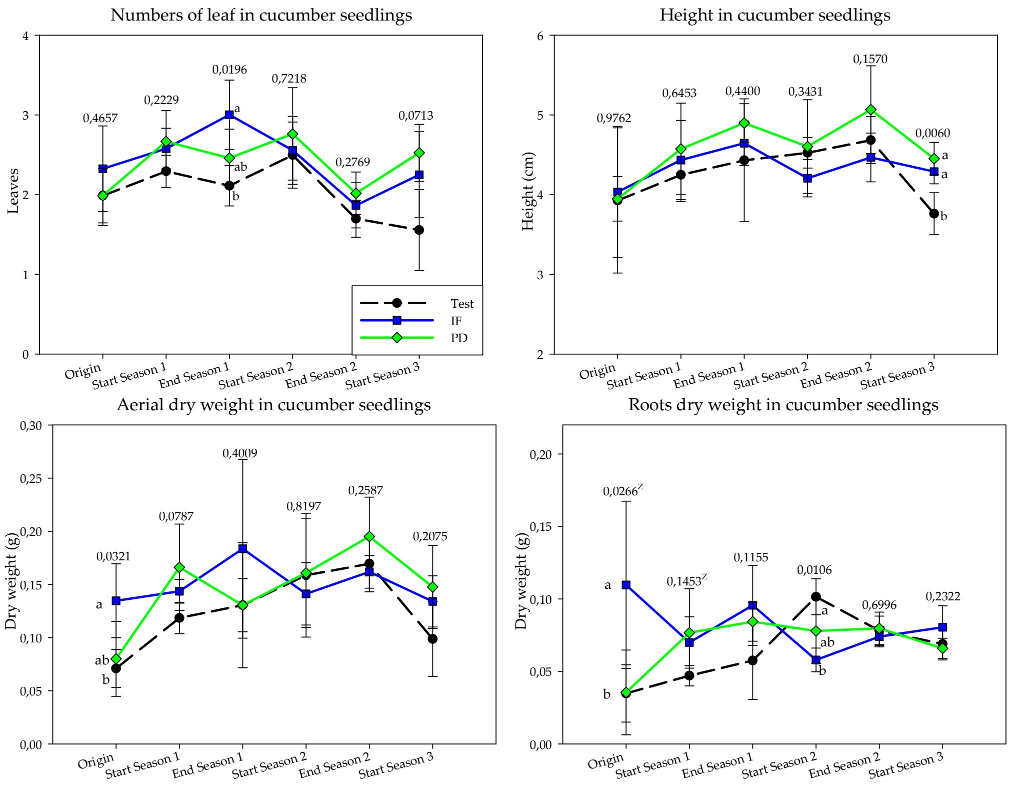

Figure S2. Number of leaves, height and root and aerial dry weight of cucumber seedlings grown in controlled chamber conditions in the three years of study (September-April cycles) as a function of crop nutrition. Tomato. Inorganic fertilization (IF; n=4); tomato plant debris (PD; n=4); no fertilization (Test; n=4). Values (mean ± standard deviation). Different letters indicate significant differences ($p \le 0.05$, Tukey's HDS test; ^{Z}:$\sqrt{x}$).

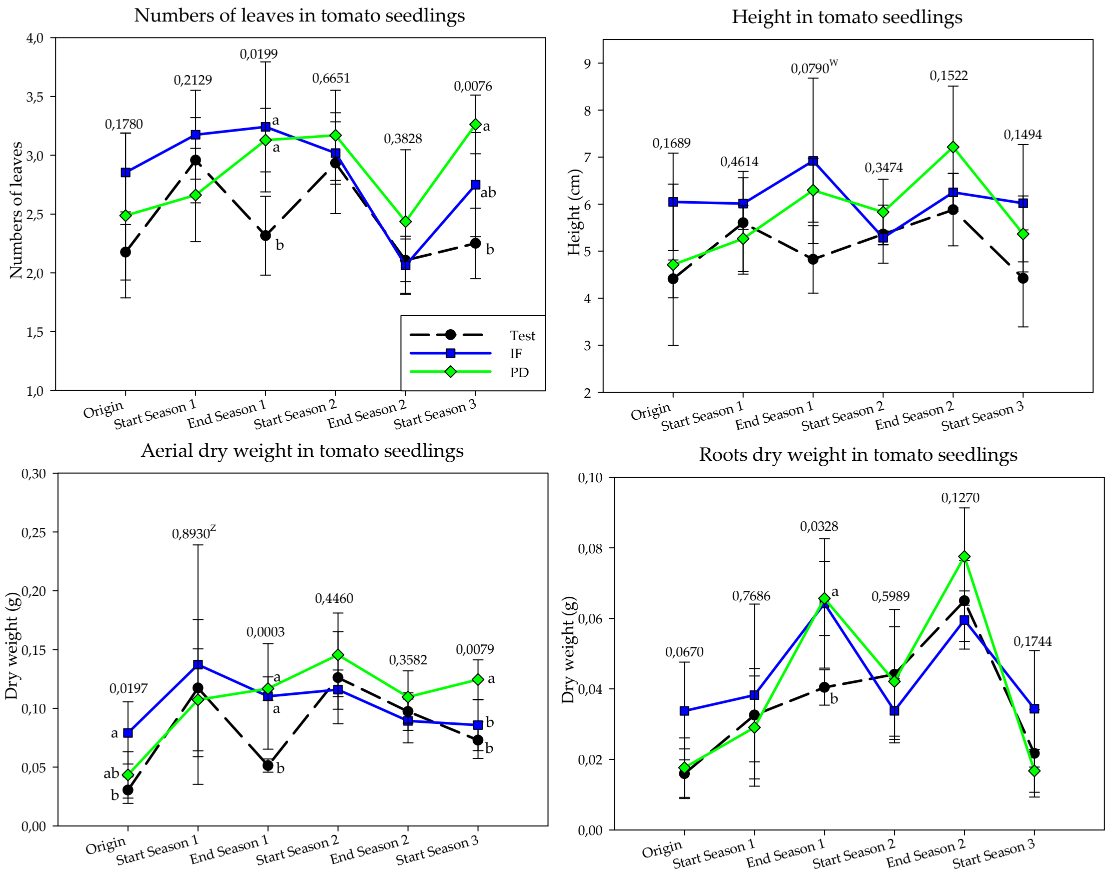

Figure S3. Number of leaves, height and root and aerial dry weight of tomato seedlings grown in controlled chamber conditions in the three years of study (September-April cycles) as a function of crop nutrition. Tomato. Inorganic fertilization (IF; n=4); tomato plant debris (PD; n=4); no fertilization (Test; n=4). Values (mean ± standard deviation). Different letters indicate significant differences (p≤0.05, Tukey's HDS test; ^{W}: $\frac{1}{\log(x)}$; ^{Z}: $\sqrt{x}$).
